# Competition between Nagaoka ferromagnetism and superconducting pairing in hybrid quantum dots

**DOI:** 10.1038/s41598-025-10867-5

**Published:** 2025-07-14

**Authors:** Emil Siuda, Ireneusz Weymann

**Affiliations:** Institute of Spintronics and Quantum Information, Faculty of Physics and Astronomy, Adam Mickiewicz University, ul. Uniwersytetu Poznańskiego 2, 61-614 Poznań, Poland

**Keywords:** Hybrid system, Nagaoka ferromagnetism, Quantum dot, Nanoscale devices, Condensed-matter physics

## Abstract

We examine the interplay between the Nagaoka ferromagnetism and superconducting pairing correlations in a two-by-two quantum dot array proximitized by an *s*-wave superconductor. Focusing on the subgap regime, we determine the phase diagram of the system, demonstrating that Nagaoka ferromagnetism becomes greatly affected by the presence of superconductor and, depending on the strength of direct and crossed Andreev reflections, can be fully suppressed. This happens at some critical value of the induced on-dot pairing potential, for which we provide the corresponding analytical formulas in the limit of large Coulomb correlations. Moreover, we also shed light on the competition between Nagaoka ferromagnetism and superconductivity visible in the transport characteristics. Using the real-time diagrammatic technique, we determine the nonequilibrium current and differential conductance assuming that the quantum dot plaquette is weakly coupled to external lead that can be used to probe the system’s properties. We not only uncover the impact of Nagaoka ferromagnetism on the Andreev current and differential conductance, but also demonstrate a spin quintuplet blockade of the Andreev transport.

## Introduction

The Hubbard model was introduced by John Hubbard in the sixties to describe the itinerant magnetism of transition and rare-earth metals^1^. Despite its simplicity, Hubbard model and its derivatives find applications for studying complex phenomena arising from strong interactions of electrons: Mott transition^2^, superconductivity^3–5^, charge and spin density waves^6–8^, topological phases^9,10^ and exotic forms of magnetism^11–18^. The experimental realizations of the fermionic Hubbard model with different geometries include, among others, optical lattices^19–22^, Moiré superlattices^23,24^ and quantum dots^25–27^. Interestingly, although intended to describe ferromagnetism, the ground state of the Hubbard model was shown to be ferromagnetic only in very limited cases^11,28–33^. One of them is the so-called *Nagaoka ferromagnetism*^11^. Nagaoka proved rigorously that under the condition of infinitely strong on-site Coulomb repulsion, with the presence of exactly one hole in an otherwise half-filled band, the spins align in parallel due to the quantum interference between different paths the hole may take when tunneling between the sites^11^.

Due to its stringent constraints, Nagaoka ferromagnetism was considered an important but rather purely theoretical result for a long time. The condition of exactly one hole present is unattainable in the thermodynamic limit, while the requirement of infinitely strong Coulomb repulsion is unphysical in any system. However, this situation has changed with the advent of nanotechnology. In quantum dot systems, the filling of each site can be controlled with gate voltages, giving an unprecedented platform to study the Hubbard model far from the thermodynamic limit^34–38^. Quantum dot arrays have already been used to realize the Mott insulator^25^ and, very recently, to prove the existence of Nagaoka ferromagnetism in experimental settings^26^. Moreover, theoretical studies found the ground state of this system to be ferromagnetic even for a finite value of the on-dot Coulomb repulsion^39^. This magnetic behavior extends to three sites arranged in a triangular structure^40^ and becomes even more complex in multilayered plaquettes^41^. Furthermore, the signatures of Nagaoka ferromagnetism were also found in the spin-polarized transport properties of the system^42^.

Lately, there has been significant interest in the investigation of various hybrid nanostructures, in which ferromagnetism competes with superconducting correlations^43–46^. In particular, when a quantum dot is placed in the proximity of a superconductor, the *Andreev bound states* form due to multiple Andreev reflections^47–52^. These states have energy within the range of the superconducting energy gap and can be a source of many remarkable transport phenomena^53^. Andreev bound states have been shown to produce Majorana quasiparticles in hybrid systems consisting of bulk superconductor and quantum dots^54–56^, dimers^57^, nanowires^58,59^ and magnetic islands^60,61^. Quantum dots and nanotubes coupled to superconducting and metallic leads were proposed as Cooper-pair splitters^62,63^ and non-local thermoelectric devices^64–66^. Although most of these studies concerned the case where the source of ferromagnetism was associated with the electrodes, much less is known about the interplay of superconducting pairing with more exotic forms of ferromagnetism, such as the one predicted by Nagaoka, emerging in coupled quantum dot systems^67^.

**Figure 1:**
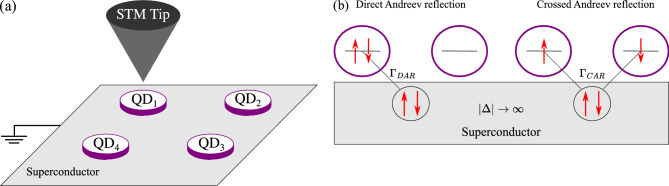
(**a**) The schematic of the considered system, consisting of four quantum dots arranged in a square plaquette placed on an *s*-wave superconducting substrate. Each quantum dot hosts a single orbital level of energy $\varepsilon _j$ and Coulomb correlations *U*, and there is a finite hopping amplitude between the quantum dots denoted by *t*. This setup can be probed by the scanning tunneling spectroscopy. (**b**) displays schematically the direct and crossed Andreev processes relevant for transport in the studied setup.

Motivated by recent experimental and theoretical results, in this paper we therefore examine the interplay between Nagaoka ferromagnetism and superconductivity in a square (two-by-two) quantum dot plaquette subject to the superconducting proximity effect, see Fig. 1a. Our considerations are performed in the limit when the superconducting energy gap is the largest energy scale and transport occurs exclusively due to Andreev reflection processes. First, we explore the phase diagram of the system depending on the strength of the on-dot Coulomb interactions and the superconducting pairing correlations associated with the direct (DAR) and crossed (CAR) Andreev reflections. Then, assuming that the proximitized quantum dot plaquette is weakly coupled to external contact, we examine the transport characteristics by employing the real-time diagrammatic technique^68^. In particular, we analyze the behavior of the current and the corresponding differential conductance in the nonlinear response regime, revealing various Andreev bound states and discussing their role in the transport characteristics.

## Model

We consider a system consisting of four quantum dots placed on an s-wave superconducting substrate, as shown in Fig. 1a. The quantum dots are arranged in a square plaquette. Moreover, each quantum dot is assumed to host a single orbital level. In addition, there may be metallic contact (or a tip of an STM) attached to one of the quantum dots, allowing for performing the Andreev transport spectroscopy.

### Hamiltonian

The considered system can be described by the following Hamiltonian

1 $$\begin{aligned} H = H_{\textrm{QDs}} + H_{\textrm{Lead}} + H_{\textrm{SC}} + H_{\textrm{TL}} + H_{\textrm{TSC}}, \end{aligned}$$

where the first term is of the form

2 $$\begin{aligned} H_{\textrm{QDs}} = \sum _{i}\big (\varepsilon _{i}\sum _\sigma d^{\dagger }_{\sigma i}d_{\sigma i} + U_{i}n_{\uparrow i}n_{\downarrow i}\big ) - \sum _{\left<ij\right>}\sum _{\sigma } \frac{t_{ij}}{2}d^{\dagger }_{\sigma i}d_{\sigma j}, \end{aligned}$$

and describes the four quantum dots, labeled by indices *i* and *j*. The energy level of quantum dot *i* is denoted by $\varepsilon _{i}$ and electrons occupying the same dot interact electrostatically with the strength described by the Hubbard parameter $U_{i}$. The hopping between the neighboring quantum dots is modeled by $t_{ij}$ and we included 1/2 for double counting. The corresponding creation and annihilation operators for electron on quantum dot *i* with spin $\sigma$ are denoted by $d^{\dagger }_{\sigma i}$ and $d_{\sigma i}$, respectively. In the following, without loss of generality, we assume identical Hubbard parameters $U_{i} \equiv U$ and hopping integrals $t_{ij}\equiv t$. Moreover, we assume that the energy levels of the quantum dots are all equal, $\varepsilon _i\equiv \varepsilon _d$. In addition, we consider the case when the capacitive coupling between the quantum dots is negligible.

The second term of the Hamiltonian describes the normal contacts, labeled by $\alpha$, attached to the quantum dots modeled as noninteracting quasiparticles. It is given by

3 $$\begin{aligned} H_{\textrm{Lead}} = \sum _{\alpha }\sum _{{\textbf{k}}\sigma }\varepsilon _{\alpha {\textbf{k}}}c^{\dagger }_{\alpha {\textbf{k}}\sigma }c_{\alpha {\textbf{k}}\sigma }, \end{aligned}$$

where $\varepsilon _{\alpha {\textbf{k}}}$ is the energy of an electron with wave vector ${\textbf{k}}$ and spin $\sigma$ in the lead $\alpha$, while $c^{\dagger }_{\alpha {\textbf{k}}\sigma }$ is the corresponding creation operator. The third term describes the *s*-wave superconductor and is given by the mean-field BCS Hamiltonian

4 $$\begin{aligned} H_{\textrm{SC}} = \sum _{{\textbf{q}}\sigma }\varepsilon _{{\textbf{q}}}s^{\dagger }_{{\textbf{q}}\sigma }s_{{\textbf{q}}\sigma } +\Delta \sum _{{\textbf{q}}}s_{{\textbf{q}}\downarrow }s_{-{\textbf{q}}\uparrow } + \Delta ^*\sum _{{\textbf{q}}}s_{-{\textbf{q}}\uparrow }^\dagger s_{{\textbf{q}}\downarrow }^\dagger , \end{aligned}$$

where $s^{\dagger }_{{\textbf{q}}\sigma }$ creates a spin-$\sigma$ particle with wave vector ${\textbf{q}}$ of energy $\varepsilon _{{\textbf{q}}}$, while $\Delta$ denotes the superconducting order parameter.

The fourth term represents the tunneling between the corresponding quantum dots and the leads. It acquires the general form

5 $$\begin{aligned} H_{\textrm{TL}} = \sum _{\alpha i}\sum _{{\textbf{k}} \sigma }V^{\alpha i}_{{\textbf{k}} \sigma }c^{\dagger }_{\alpha {\textbf{k}}\sigma }d_{i\sigma }+\mathrm {H.c.}, \end{aligned}$$

where $V^{\alpha i}_{{\textbf{k}} \sigma }$ are the tunneling matrix elements between the $\alpha$-th lead and the *i*-th quantum dot. We assume the tunneling to be spin and momentum-independent, $V^{\alpha i}_{{\textbf{k}} \sigma } \equiv V_{\alpha i}$, which allows us to write the coupling strength between the dot *i* and the lead $\alpha$ as, $\Gamma _i^{\alpha } = 2\pi |V_{\alpha i}|^2 \rho _{\alpha }$, where $\rho _\alpha$ is the density of states of the lead $\alpha$. Here, we will focus on the case when the system is probed by a tip of STM, as schematically depicted in Fig. 1a, therefore, $\Gamma _i^{\alpha }\equiv \Gamma$.

Finally, the last term of the total Hamiltonian has a similar form and describes the tunneling between the superconductor and the quantum dots

6 $$\begin{aligned} H_{\textrm{TSC}} = \sum _{i}\sum _{{\textbf{q}} \sigma } V^{S i}_{{\textbf{q}} \sigma }s^{\dagger }_{{\textbf{q}}\sigma }d_{i\sigma }+\mathrm {H.c.}, \end{aligned}$$

where $V^{S i}_{{\textbf{q}} \sigma }$ are the corresponding tunneling matrix elements, which give rise to the quantum dot-superconductor hybridization $\Gamma ^{S}_{i} = 2\pi |V_{S i}|^2 \rho _S$, with $\rho _S$ being the density of states of a superconductor in the normal state.

In the following, we focus on the regime where the Andreev bound states form in the quantum dot plaquette, interplaying with the Nagaoka ferromagnetism, and transport between the superconductor and quantum dots takes place exclusively via Andreev reflections. We therefore consider the case where $\Delta$ is the largest energy scale present in the system and employ the so-called atomic limit. Under such an approximation, the quantum dots coupled to the superconductor can be modeled by an effective Hamiltonian, ${H_{\textrm{QDs}}^{\textrm{eff}} = H_{\textrm{QDs}} + H_{\textrm{SC}} + H_{\textrm{TSC}}}$, the microscopic form of which reads^69^

7 $$\begin{aligned} H_{\textrm{QDs}}^\textrm{eff} = H_{\textrm{QDs}} - \frac{1}{2}\sum _{i}\Gamma ^{S}_{i} \big ( d^{\dagger }_{\uparrow i}d^{\dagger }_{\downarrow i} + \mathrm {H. c.}\big ) + \frac{1}{2} \sum _{\left<ij\right>} \frac{\Gamma ^{S}_{ij}}{2} \big ( d^{\dagger }_{\uparrow i}d^{\dagger }_{\downarrow j} + d^{\dagger }_{\uparrow j}d^{\dagger }_{\downarrow i} + \mathrm {H. c.}\big ). \end{aligned}$$

The two emergent terms describe proximity-induced Andreev reflections of electrons between quantum dots and the superconducting substrate. The first one, proportional to $\Gamma ^{S}_{i}$, corresponds to direct Andreev reflections, in which the Cooper pair tunnels to or from the same quantum dot. The second term, proportional to $\Gamma ^{S}_{ij}$, is associated with crossed Andreev reflections, in which electrons forming a Cooper pair split upon entering two different quantum dots or form a Cooper pair when they tunnel from neighboring dots to the superconductor. Again, the factor of 1/2 is included to avoid double counting. Both types of Andreev reflections are schematically depicted in Fig. 1b. In general, the value of the coupling between each dot and the superconductor can differ, here, however, for the sake of clarity, we assume the symmetric case, $\Gamma ^{S}_i\equiv \Gamma _{\textrm{DAR}}$ and $\Gamma ^{S}_{ij} \equiv \Gamma _{\textrm{CAR}}$.

### Quantities of interest

One of the objectives of this work is to find the signatures of Nagaoka ferromagnetism and its interplay with superconducting pairing in the Andreev transport properties of the system. To determine transport characteristics, we employ the real-time diagrammatic technique^68,70,71^, which allows for calculating the transport quantitites order by order in tunneling processes. We define $I_{\alpha }$ as the current flowing between the $\alpha$-th lead and the corresponding quantum dot connected to it, and $I_{\textrm{S}}$ as the Andreev current flowing between the superconductor and the quantum dots. Then, the Andreev current can generally be found from the current conservation, $I_{\textrm{S}} = \sum _\alpha I_{\alpha }$. After calculating the currents, the Andreev differential conductance can be found from $G_{\textrm{S}} = dI_{\textrm{S}}/dV$. More details about the specific calculations are given in the Methods section.

## Results

In the following, we first present and discuss the relevant phase diagrams of the system, where we briefly introduce the Nagaoka ferromagnetism for isolated quantum dot plaquette and then study the influence of the superconducting proximity effect. In the next step, we explore and analyze the transport properties of this hybrid system.

### Isolated quantum dot plaquette

To begin with, let us consider the situation when the quantum dot plaquette is isolated^39^, i.e. it is neither coupled to external normal leads nor to the superconductor. In this case, restricting to the statespace with 3 electrons on the quadruple quantum dot, one can determine the eigenenergies exactly. For the lowest-energy state in the spin quadruplet $S=3/2$ subspace, one finds $E_{3/2} = 3\varepsilon _d -2 t$. On the other hand, the lowest-energy doublet state has the energy

8 $$\begin{aligned} E_{1/2} = 3\varepsilon _d+\frac{U}{2} - \frac{1}{2} \sqrt{32 t^2 + U^2 + 4 t \sqrt{64 t^2+3U^2}}. \end{aligned}$$

Then, it is straightforward to find a critical value of the Coulomb correlations $U_{\textrm{crit}}$, for which the ground state changes from the doublet to the quadruplet state. This happens exactly when

9 $$\begin{aligned} U_{\textrm{crit}}= 4 (2 + \sqrt{7}) t, \end{aligned}$$

which gives $U_{\textrm{crit}}/t \approx 18.583$. Note that this value does not depend on $\varepsilon _d$ as long as the quantum dot system is occupied by three electrons. In other words, in the case of infinite Coulomb correlations, the ground state is always ferromagnetic, while the Nagaoka ferromagnetism ceases to exist once $U < U_{\textrm{crit}}$. This picture will be modified by the superconducting correlations, as discussed in the sequel. One also needs to keep in mind that, decreasing the correlations much beyond $U_{\textrm{crit}}$, the occupation of the quantum dot plaquette eventually changes to even, and the ground state becomes spin singlet $S=0$.

### Phase diagram in the presence of superconducting proximity effect

In the presence of the superconducting proximity effect, the analytical considerations are much more cumbersome, and it is not possible to find reasonably simple analytical formulas for the eigenenergies of the system in the general case. This is because now the total particle number *N* of the hybrid quantum dot system is no longer a good quantum number and the Hamiltonian only conserves the total spin *S* and its *z*-th component $S_z$. Therefore, we will resort to numerical calculations, while analytical formulas will be provided in the limit of large Coulomb correlations. In particular, we calculate the dependence of the expectation value of $\langle N \rangle$ and determine the spin quantum number *S* of the ground state as a function of *U*/*t* and the superconducting pairing correlations. To find the occupation $\langle N \rangle$, we perform numerical diagonalization of the Hamiltonian (7), which allows us to set up the density matrix and determine the expectation values of interest. In calculations, we use $t\equiv 1$ as the energy unit and assume infinitesimal temperature on the order of $T=10^{-8}$ to extract the ground state properties. To differentiate between the influence of particular Cooper pair’s tunneling processes, we perform calculations taking into account each of them separately, and with both CAR and DAR present with different tunneling amplitudes.

#### The role of DAR and CAR processes

**Figure 2:**
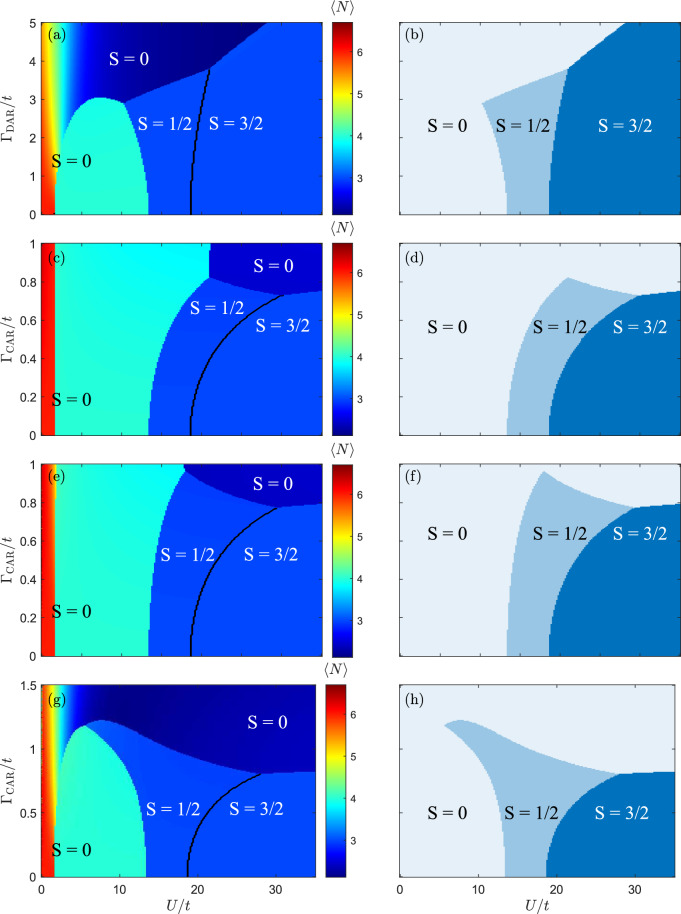
The ground state phase diagram of the considered system as a function of the Coulomb correlation parameter *U* and the superconducting pairing potential when: (**a**,**b**) $\Gamma _{\textrm{DAR}} \ne 0$ and $\Gamma _{\textrm{CAR}} = 0$, (**c**,**d**) $\Gamma _{\textrm{CAR}}\ne 0$ and $\Gamma _{\textrm{DAR}} = 0$, (**e**,**f**) $\Gamma _{\textrm{DAR}} = \Gamma _{\textrm{CAR}}$, and (**g**,**h**) $\Gamma _{\textrm{DAR}} = 2\Gamma _{\textrm{CAR}}$. The left column presents the expectation value of the quadruple quantum dot occupation $\langle N \rangle$, while the right column displays the spin quantum number *S* of the ground state. The other parameters are: $\varepsilon _d/t = -1.25$ and $t \equiv 1$ is used as the energy unit.

In order to analyze the effect of DAR and CAR processes on the Nagaoka ferromagnetism, we tune the system to the transport regime where this type of ferromagnetism can emerge in the absence of superconductor. Then, for vanishing $\Gamma _{\textrm{CAR}}$ and $\Gamma _{\textrm{DAR}}$, one should observe the development of Nagaoka ferromagnetism signaled by the formation of $S=3/2$ ground state for $U>U_{\textrm{crit}}\approx 18.583 \; t$. Moreover, for assumed value of $\varepsilon _d/t = -1.25$, there is another change of ground state for $U\approx 13.424\;t$, where the occupation switches to $\langle N \rangle =4$ and the ground state becomes the spin singlet $S=0$. Finally, the occupation changes to $\langle N \rangle =6$ once $U\lesssim 1.58\;t$. All these different phases can be clearly recognized in Fig. 2 in the case of $\Gamma _{\textrm{CAR}}=\Gamma _{\textrm{DAR}}=0$. This figure presents the dependence of $\langle N \rangle$ and *S* on *U*/*t* and the proximitiy-induced pairing, assuming different ratios of the DAR and CAR processes. Generally, Nagaoka ferromagnetism prevails independent of the type of Cooper pair tunneling, but only up to some critical value of the coupling strength. In each case, the critical value of the Hubbard parameter, for which the transition between antiferromagnetic and ferromagnetic states occurs, depends on the value of the on-dot pairing. However, when the superconducting pairing is sufficiently strong, only nonmagnetic phase, is present.

To facilitate identification of different phases, we mark them in the figure by explicitly assigning the spin quantum numbers. Moreover, since the charge is not conserved due to the superconducting proximity effect, we only present the expectation value of *N* with a well-separated colorscale. One can clearly see that the magnetic phases ($S=1/2$ or $S=3/2$) develop when the occupation is odd, i.e. $\langle N \rangle \approx 3$, while nonmagnetic phases ($S=0$) are present when $\langle N \rangle$ is even. In particular, this happens in regions where $\langle N \rangle \approx 4$ or $\langle N \rangle \approx 6$ with lowering *U*/*t* and for low amplitudes of Andreev processes. On the other hand, when the superconducting pairing dominates over the hopping amplitude, nonmagnetic phase with $\langle N \rangle \approx 2$ develops, see Fig. 2.

Let us now start a more detailed discussion beginning with the case of $\Gamma _{\textrm{CAR}}\rightarrow 0$ and finite $\Gamma _{\textrm{DAR}}$, which is shown in the first row of Fig. 2. This situation is relevant for systems where quantum dots are spatially well-separated, or when the superconducting phase coherence length is short, such that crossed Andreev reflection processes are suppressed and only direct Andreev reflections are relevant. One can see that, by increasing $\Gamma _{\textrm{DAR}}$, the critical value of the correlations, $U_{\textrm{crit}}$, for which the antiferromagnetic-ferromagnetic transition occurs, increases smoothly. On the other hand, the singlet-doublet transition occurs at lower *U* when $\Gamma _{\textrm{DAR}}$ is raised, until around $\Gamma _{\textrm{DAR}}\approx 3\; t$, when the system ground state becomes nonmagnetic, $S=0$, see Fig. 2b. The corresponding transitions are also revealed in the expectation value of the quantum dot occupation, where the regions of approximate even and odd $\langle N \rangle$ are clearly visible, and separate the respective different spin ground states.

The phase diagram becomes modified when only crossed Andreev reflections are allowed, which happens in systems with large Coulomb correlations^72,73^, prohibiting double occupancy of individual quantum dots, see Fig. 2c,d. Now, one can see that CAR processes affect the magnetic phases more strongly, i.e. both the spin doublet and quadruplet phases persist up to $\Gamma _{\textrm{CAR}}\approx 3t/4$, while for larger superconducting pairing only the singlet phase is relevant. This is strictly associated with the type of Andreev reflection processes. In CAR tunneling, the Cooper pair is split between different dots, enhancing the antiferromagnetic correlations between them. This effectively reduces the phase space in which Nagaoka ferromagnetism can exist. When enhancing $\Gamma _{\textrm{CAR}}$ for $U>U_{\textrm{crit}}$, one can also see that a change of $\langle N \rangle$ from odd to even occurs, giving rise to a nonmagnetic phase.

The case when both DAR and CAR processes are relevant, and contribute on an equal footing, is presented in Fig. 2e,f. In this situation, the general behavior is very similar to the case with only CAR processes present. The main difference is associated with a slight change in the value of $\Gamma _{\textrm{CAR}}$, at which the transition between the ferromagnetic and antiferromagnetic phases occurs. It is also worth noting that now the doublet phase is slightly enlarged compared to the previous cases, which is associated with the fact that the presence of DAR processes reduces the role of antiferromagnetic correlations triggered by crossed Andreev reflections.

In typical experiments, the rate of crossed Andreev reflection processes may be smaller than that of direct Andreev processes, which is due to a certain distance between the neighboring quantum dots. To address this case, we study the behavior of the system assuming $\Gamma _{\textrm{DAR}}= 2\Gamma _{\textrm{CAR}}$. The resulting phase diagram is shown in the lowest row of Fig. 2. One can see that for larger values of *U*, see $U\gtrsim 25 \; t$ in Fig. 2h, the transition between the states $S=3/2$ and $S=0$ occurs at similar values of $\Gamma _{\textrm{CAR}}$ as compared to previous situations. Consequently, Nagaoka ferromagnetism is robust against superconducting correlations unless $\Gamma _{\textrm{CAR}}\gtrsim 3t/4$. Moreover, there is a clear nonmonotonic dependence of the ground state on $\Gamma _{\textrm{CAR}}$ for *U* slightly smaller than the singlet-doublet transition in the case of $\Gamma _{\textrm{CAR}}=0$, see the case of $U\approx 11 \; t$ in Fig. 2h. As can be seen in the figure, the superconducting correlations can induce a doublet phase in a certain range of parameters. This is also associated with a change of $\langle N \rangle$ to odd occupation; see Fig. 2g for $\Gamma _{\textrm{CAR}}\approx t$ and $U\approx 10\; t$.

As results from the above analysis, the Andreev reflection processes have generally detrimental effect on the ferromagnetic correlations in the system. Large enough Andreev processes suppress the Nagaoka ferromagnetism and induce a BCS singlet phase of the system. While on-dot pairing (DAR processes) favor BCS singlet in each quantum dot, inter-dot pairing (CAR processes) give rise to formation of singlets between neighboring quantum dots. Consequently, when both DAR and CAR processes are present in the system, one observes a complex phase diagram with different magnetic and nonmagnetic phases (see Fig. 2), with nonmagnetic phase generally signaling a crucial role of the Andreev reflection processes.

#### The case of large Coulomb correlations

**Figure 3:**
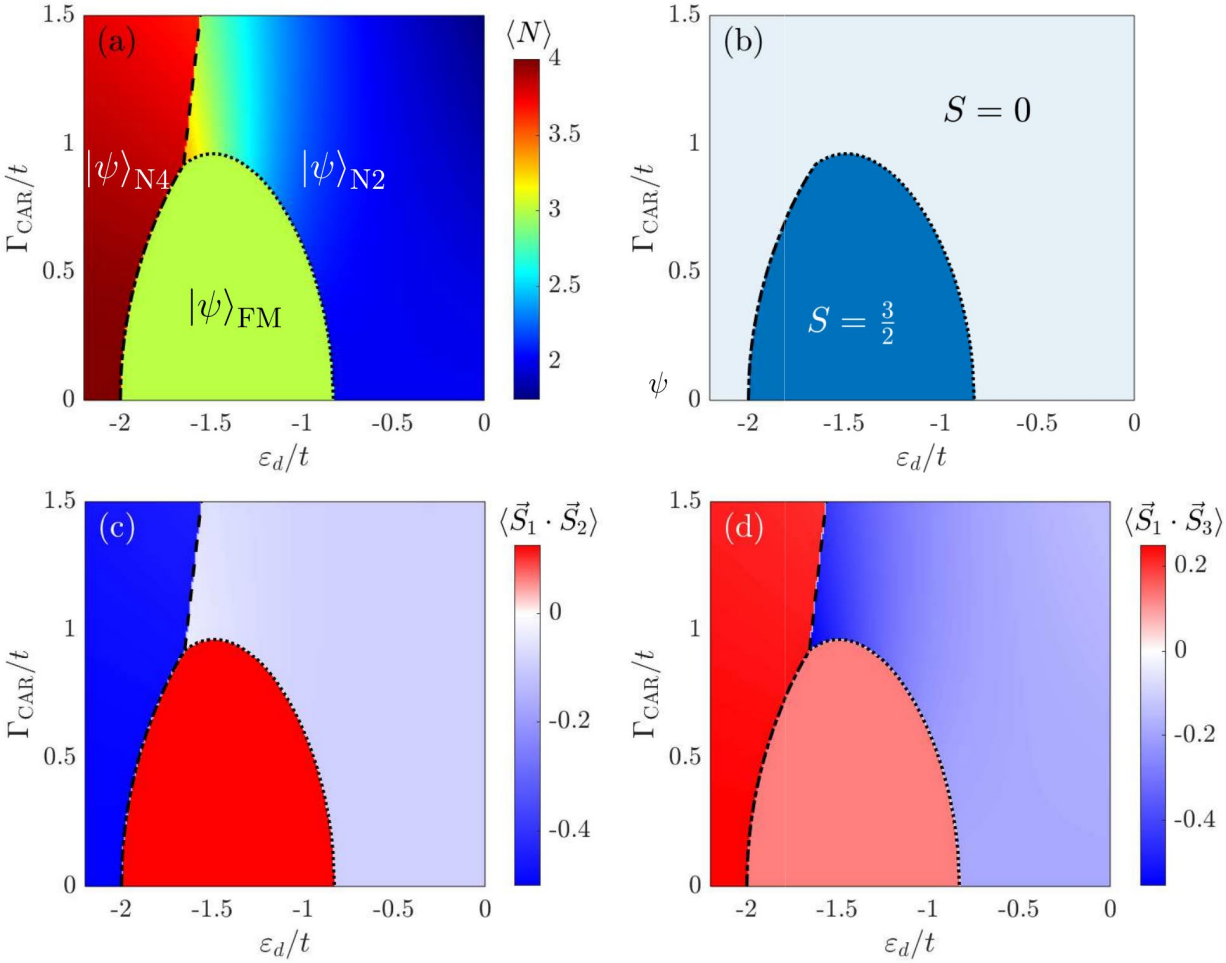
The phase diagram of the system (top row) and expectation values of correlations between electron spins occupying different dots (bottom row) as a function of the quantum dot energy level $\varepsilon _d$ and the crossed Andreev reflection pairing $\Gamma _{\textrm{CAR}}$ in the case of infinite Coulomb correlations ($\Gamma _{\textrm{DAR}}=0$). Panel (**a**) presents the behavior of the particle number expectation value, while panel (**b**) displays the ground state spin quantum number. Panel (**c**) presents the expectation value of (**c**) spins of neighboring quantum dots $\langle \textbf{S}_1 \cdot \textbf{S}_2 \rangle$ and (**d**) spins of next-neighbor quantum dots $\langle \textbf{S}_1 \cdot \textbf{S}_3 \rangle$. The hopping parameter is taken as the energy unit $t \equiv 1$.

To obtain some analytical results and gain more insight into the physics of the system, we now focus on the regime where the double occupancy of quantum dots is negligible. In this case, DAR processes are suppressed, and the behavior of the system is governed by the interplay between the inter-dot hopping and crossed Andreev reflections. Such regime is frequently relevant for hybrid quantum dots that are basic building blocks for the minimal Kitaev chain, in which the physics is mostly governed by cotunneling and CAR processes^72,73^. The phase diagram in the case of negligible DAR processes is presented in Fig. 3. This figure shows the expectation value of occupation $\langle N \rangle$ and the spin *S* of the ground state, together with the expectation values of the correlations between the spins of the electrons occupying neighboring dots, $\langle \textbf{S}_1 \cdot \textbf{S}_2 \rangle$ and the next-neighboring dots $\langle \textbf{S}_1 \cdot \textbf{S}_3 \rangle$, as a function of the energy level of the quantum dot $\varepsilon _d$ and the crossed Andreev reflection pairing $\Gamma _{\textrm{CAR}}$. As can be seen in Fig. 3b, in the ground state, the system can be either ferromagnetic with $S = 3/2$ or non-magnetic with $S = 0$. Due to the infinite Hubbard parameter, the antiferromagnetic state is no longer the ground state for any values of the parameters. Moreover, now, one clearly sees that in the $S=3/2$ ground state, the spin exchange interactions between the neighbor and next-nearest neighbor quantum dots are both ferromagnetic, $\langle \textbf{S}_i \cdot \textbf{S}_j \rangle >0$, see Fig. 3c,d. On the other hand, in the singlet ground state, $\langle \textbf{S}_i \cdot \textbf{S}_j \rangle <0$ for $\varepsilon _d/t \gtrsim -1.5$, while for $\varepsilon _d/t \lesssim -1.5$ the neighboring dot spins tend to interact antiferromagnetically, while the next-nearest neighbor interactions are ferromagnetic. The change of the exchange interactions is associated with a change of occupation from $\langle N \rangle \approx 2$ to $\langle N \rangle \approx 4$ as the level position is lowered, see Fig. 3a. The corresponding phase boundaries are marked in the figure with dotted and dashed lines. Moreover, we also mark the states that have the highest occupation probability in the corresponding phases.

To obtain some analytical estimates for the phase diagram, let us focus on the Hamiltonian blocks corresponding to the quadruplet and singlet subspaces. Moreover, we exploit the spin *SU*(2) symmetry to reduce the size of the Hilbert space. The Hilbert subspace corresponding to the highest-weight component of $S=3/2$ is spread over four states

10 $$\begin{aligned} {\mathscr {H}}_{S=\frac{3}{2}} = \left\{ {|{\uparrow \uparrow \uparrow 0}\rangle },{|{\uparrow \uparrow 0\uparrow }\rangle },{|{\uparrow 0\uparrow \uparrow }\rangle },{|{0\uparrow \uparrow \uparrow }\rangle }\right\} , \end{aligned}$$

with the corresponding block Hamiltonian

11 $$\begin{aligned} H_{S=\frac{3}{2}} = \begin{bmatrix} 3\varepsilon _d& -t& 0& -t\\ -t& 3\varepsilon _d& -t& 0\\ 0& -t& 3\varepsilon _d& -t\\ -t& 0& -t& 3\varepsilon _d \end{bmatrix}. \end{aligned}$$

The ground state of this Hamiltonian is $${|{\psi }\rangle }_{\textrm{FM}} = \frac{1}{2}\begin{bmatrix} 1&1&1&1 \end{bmatrix}^T$$ and has the energy $E_{\textrm{FM}} = 3\varepsilon _d-2t$. Similarly, as for finite on-dot Coulomb interaction, the system exhibits Nagaoka ferromagnetism up to some critical value of the hopping *t* dependent on the dot’s level position.

On the other hand, the spin-singlet Hilbert space can be constructed from the following representative states

12 $$\begin{aligned} {\mathscr {H}}_{S=0} =\left\{ {|{0000}\rangle },{|{\uparrow \downarrow 00}\rangle },{|{00\uparrow \downarrow }\rangle },{|{\uparrow 0\downarrow 0}\rangle },{|{0\uparrow \downarrow 0}\rangle }, {|{\uparrow 00\downarrow }\rangle },{|{0\uparrow 0\downarrow }\rangle },{|{\uparrow \downarrow \uparrow \downarrow }\rangle },{|{\uparrow \uparrow \downarrow \downarrow }\rangle } \right\} , \end{aligned}$$

while the Hamiltonian in this subspace is explicitly given by

13 $$\begin{aligned} H_{S=0} = \begin{bmatrix} 0& \frac{\Gamma _{\textrm{CAR}}}{\sqrt{2}}& \frac{\Gamma _{\textrm{CAR}}}{\sqrt{2}}& 0& \frac{\Gamma _ {\textrm{CAR}}}{\sqrt{2}}& \frac{\Gamma _{\textrm{CAR}}}{\sqrt{2}}& 0& 0& 0\\ \frac{\Gamma _{\textrm{CAR}}}{\sqrt{2}}& 2\varepsilon _d& 0& -t& 0& 0& -t& \frac{\Gamma _{\textrm{CAR}}}{\sqrt{2}}& 0\\ \frac{\Gamma _{\textrm{CAR}}}{\sqrt{2}}& 0& 2\varepsilon _d& -t& 0& 0& -t& \frac{\Gamma _{\textrm{CAR}}}{\sqrt{2}}& 0\\ 0& -t& -t& 2\varepsilon _d& -t& -t& 0& 0& 0\\ \frac{\Gamma _{\textrm{CAR}}}{\sqrt{2}}& 0& 0& -t& 2\varepsilon _d& 0& -t& -\frac{\Gamma _{\textrm{CAR}}}{2\sqrt{2}}& \sqrt{\frac{3}{8}}\Gamma _{\textrm{CAR}}\\ \frac{\Gamma _{\textrm{CAR}}}{\sqrt{2}}& 0& 0& -t& 0& 2\varepsilon _d& -t& -\frac{\Gamma _{\textrm{CAR}}}{2\sqrt{2}}& \sqrt{\frac{3}{8}}\Gamma _{\textrm{CAR}}\\ 0& -t& -t& 0& -t& -t& 2\varepsilon _d& 0& 0\\ 0& \frac{\Gamma _{\textrm{CAR}}}{\sqrt{2}}& \frac{\Gamma _{\textrm{CAR}}}{\sqrt{2}}& 0& -\frac{\Gamma _ {\textrm{CAR}}}{2\sqrt{2}}& -\frac{\Gamma _{\textrm{CAR}}}{2\sqrt{2}}& 0& 4\varepsilon _d& 0\\ 0& 0& 0& 0& \sqrt{\frac{3}{8}}\Gamma _{\textrm{CAR}}& \sqrt{\frac{3}{8}}\Gamma _{\textrm{CAR}}& 0& 0& 4\varepsilon _d\\ \end{bmatrix}. \end{aligned}$$

The ground state of this block depends on the value of the dots’ energy level and the crossed-Andreev reflection coupling and is a complex superposition of the following six states: ${|{\uparrow \downarrow 00}\rangle },{|{00\uparrow \downarrow }\rangle }$, ${|{0\uparrow \downarrow 0}\rangle },{|{\uparrow 00\downarrow }\rangle }$, ${|{\uparrow \downarrow \uparrow \downarrow }\rangle },{|{\uparrow \uparrow \downarrow \downarrow }\rangle }$. The occupations of these states in the absence of superconductor are $N = 4$ and $N = 2$, therefore, we denote the relevant states as ${|{\psi }\rangle }_{\textrm{N4}}$ and ${|{\psi }\rangle }_{\textrm{N2}}$, respectively. The regions where these states are relevant are marked in Fig. 3a. The first state turns out to be the ground state for $\varepsilon _d/t \gtrsim -1.5$, while the second state is the ground state for smaller values of orbital level energy. The unnormalized form of the eigenvector ${|{\psi }\rangle }_{\textrm{N4}}$ can be expressed as

14 $$\begin{aligned} {|{\psi }\rangle }_{\textrm{N4}} = \begin{bmatrix}&0&\frac{1}{\sqrt{1+\rho ^2}-\rho }&\frac{1}{\sqrt{1+\rho ^2}-\rho }&0&\left( \sqrt{1+\rho ^2}+\rho \right)&-\left( \sqrt{1+\rho ^2}+\rho \right)&0&-\sqrt{3}&1 \end{bmatrix}^\textrm{T}, \end{aligned}$$

where $\rho = \sqrt{\frac{2}{3}}\frac{\varepsilon _d}{\Gamma _{\textrm{CAR}}}$, and the corresponding eigenvalue is

15 $$\begin{aligned} E_{\textrm{N4}} = 3\varepsilon _d-\sqrt{\varepsilon _d^2+\frac{3\Gamma _{\textrm{CAR}}^2}{2}}. \end{aligned}$$

To better understand the results presented in Fig. 3, we calculate the eigenvector ${|{\psi }\rangle }_{\textrm{N4}}$ in the limits of $\Gamma _{\textrm{CAR}}\rightarrow 0$ and $\Gamma _{\textrm{CAR}}\rightarrow \infty$ assuming $\varepsilon _d < 0$. One then finds

16 $$\begin{aligned} \lim _{\Gamma _{\textrm{CAR}}\rightarrow 0} {|{\psi }\rangle }_{\textrm{N4}}&= \begin{bmatrix} 0&0&0&0&0&0&0&-\sqrt{3}&1 \end{bmatrix}^\textrm{T}, \end{aligned}$$

17 $$\begin{aligned} \lim _{\Gamma _{\textrm{CAR}}\rightarrow \infty } {|{\psi }\rangle }_{\textrm{N4}}&= \begin{bmatrix} 0&1&1&0&-1&-1&0&-\sqrt{3}&1 \end{bmatrix}^\textrm{T} . \end{aligned}$$

This result clearly shows that, with increasing CAR amplitude, the contribution of doubly occupied states to the superposition increases, resulting in lower mean occupation; such behavior can be seen in Fig. 3a. This is in contrast to the ferromagnetic phase, for which the contribution of each state to the superposition does not depend on the coupling to the superconductor. On the other hand, as far as the ground state for $\varepsilon _d/t \lesssim -1.5$ is considered, it is cumbersome to perform a similar analysis as before, since the coefficients of ${|{\psi }\rangle }_{\textrm{N2}}$ are complex polynomial functions of the model parameters. We performed a numerical analysis of the dependence of the particular components of the ${|{\psi }\rangle }_{\textrm{N2}}$ ground state on the dot’s energy $\varepsilon _d$ and the coupling strength $\Gamma _{\textrm{CAR}}$. It turns out that the dominant contribution comes from the local states ${|{\uparrow \downarrow \uparrow \downarrow }\rangle }$ and ${|{\uparrow \uparrow \downarrow \downarrow }\rangle }$. The contributions of other components are decreasing functions of $\varepsilon _d$, meaning that the mean occupation increases with lowering the dots’ level. Regardless of the coupling to the superconductor, close to the phase boundary between the ${|{\psi }\rangle }_{\textrm{N4}}$ and ${|{\psi }\rangle }_{\textrm{N2}}$ ground states, the main contribution comes from the state ${|{\uparrow \uparrow \downarrow \downarrow }\rangle }$ with others also contributing significantly, resulting in $\langle N \rangle \approx 3$. On the other side, far from the boundary, the dominating contribution depends on the value of the coupling strength $\Gamma _{\textrm{CAR}}$. If the coupling is strong, i.e. $\Gamma _{\textrm{CAR}}/t \gtrsim 1.4$, the ${|{0000}\rangle }$ state contributes the most resulting in the mean occupation $\langle N \rangle < 2$. For moderate values of the coupling, the states ${|{\uparrow \downarrow 00}\rangle },{|{\uparrow 0\downarrow 0}\rangle }$ and their symmetric counterparts dominate, leading to the mean occupation $\langle N \rangle \approx 2$. Despite not having exact solutions for the ${|{\psi }\rangle }_{\textrm{N2}}$ state, it is still possible to obtain explicit formulas for the phase boundaries. We indicate them with dotted (${|{\psi }\rangle }_{\textrm{N2}} \rightarrow {|{\psi }\rangle }_{\textrm{FM}}$ transition), dotted-dashed (${|{\psi }\rangle }_{\textrm{N4}} \rightarrow {|{\psi }\rangle }_{\textrm{FM}}$ transition) and dashed (${|{\psi }\rangle }_{\textrm{N2}} \rightarrow {|{\psi }\rangle }_{\textrm{N4}}$ transition) lines in Fig. 3. The formulas for the boundaries in appropriate parameter space are given in the corresponding order as

18 $$\begin{aligned} \Gamma _{\textrm{CAR}}&= \sqrt{\frac{6 \varepsilon _d^4-16 \varepsilon _d^3 t-64 \varepsilon _d^2 t^2+32 t^4}{\varepsilon _d^2+8 \varepsilon _d t-20 t^2}}, \end{aligned}$$

19 $$\begin{aligned} \Gamma _{\textrm{CAR}}&= \sqrt{\frac{8t^2 - 2\varepsilon _d^2}{3}}, \end{aligned}$$

20 $$\begin{aligned} \varepsilon _d&= -\frac{1}{8} \sqrt{\frac{3}{2}} \sqrt{\frac{64 t^4-4 t^2 \Gamma _{\textrm{CAR}}^2+3 \Gamma _{\textrm{CAR}}^2+\left( 4 t^2-\Gamma _{\textrm{CAR}}^2\right) \sqrt{\left( 256 t^4+32 t^2 \Gamma _{\textrm{CAR}}^2+9 \Gamma _{\textrm{CAR}}^4\right) }}{t^2}}. \end{aligned}$$

From the above expressions, one can find the position of the triple point of the system to be $\varepsilon _d/t \approx -1.65$ and $\Gamma _{\textrm{CAR}}/t \approx 0.92$.

### Tunneling current and differential conductance

**Figure 4:**
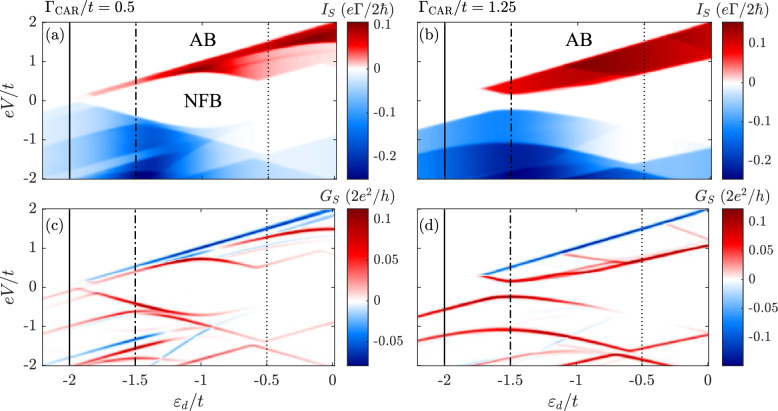
(**a**,**b**) The current $I_S$ and (**c**,**d**) the differential conductance $G_S$ in the $U \rightarrow \infty$ limit as a function of the dot’s level position $\varepsilon _d$ and the bias voltage *V* applied between the normal metal lead and superconductor calculated for the indicated values of the coupling strength $\Gamma _{\textrm{CAR}}$. The vertical lines mark the cross-sections shown in Fig. 5. The regions of Andreev blockade (AB) and blockade with Nagaoka ferromagnetism (NFB) are marked. The other parameters are: temperature $k_\textrm{B}T/t = 0.015$, coupling strength to the normal lead $\Gamma /t = 0.01$ and $t \equiv 1$ is used as an energy unit.

**Figure 5:**
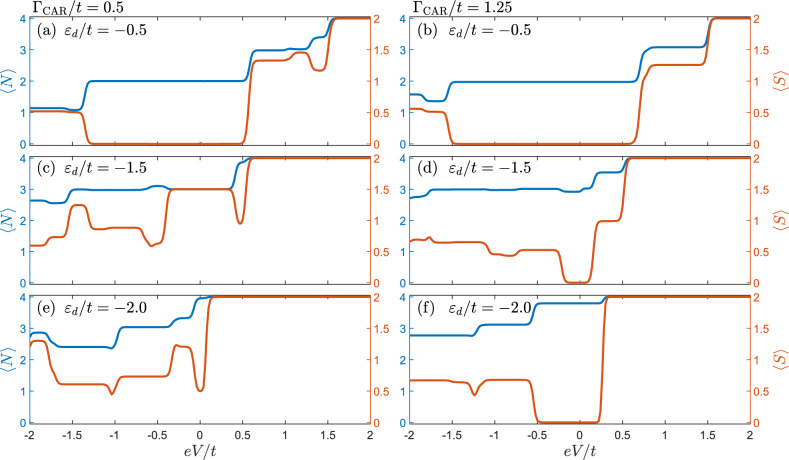
The bias voltage dependence of the expectation values of spin $\langle S \rangle$ and occupation $\langle N \rangle$ calculated for selected values of $\varepsilon _d$ and $\Gamma _{\textrm{CAR}}$, as indicated in the figure and marked in Fig. 4 with vertical lines. The other parameters are the same as in Fig. 4.

In this section, we intend to seek for the signatures of the interplay between the Nagaoka ferromagnetism and superconducting correlations in the Andreev transport properties of the system. We especially focus on the low-energy regime, where Coulomb correlations suppress the DAR processes and only crossed Andreev reflections are responsible for the Andreev current. To adapt an experimentally relevant scenario, we assume that one of the QDs is connected to the source of electrons kept at the potential *V*, while the superconductor is grounded, see Fig. 1a. The attached normal lead can thus be viewed as a tip of the STM that is used to probe the system by the bias spectroscopy. Furthermore, due to the symmetry of the system, the transport characteristics are independent of which quantum dot is attached to the normal lead. In order to examine the Andreev current and the corresponding differential conductance, we employ the real-time diagrammatic technique^68,70,71^ assuming a weak coupling to the normal lead, while the coupling to the superconducting substrate is arbitrary.

Figure 4 presents the Andreev current and differential conductance as functions of the dots’ level position $\varepsilon _d$ and the applied transport voltage *V*. We plot the calculated quantities for two values of $\Gamma _{\textrm{CAR}}$: one above and one below the critical value of the coupling for which Nagaoka ferromagnetism persists, as discussed in the previous section. In both cases, different blockade regions can be observed. In the low bias voltage regime, the main blockade mechanism is associated with the Coulomb correlations, which block transport when the bias voltage is below a certain threshold voltage. Of course, changing $\varepsilon _d$, one can tune this blockade as well as the spin state of the system, in particular, passing through the Nagaoka ferromagnetic phase. On the other hand, the blockade of the Andreev current can also occur at finite bias voltage, giving rise to negative differential conductance. Such an Andreev blockade (AB) occurs when the system becomes trapped in the highest-weight spin state, so that tunneling of Cooper pairs becomes blocked. All these types of blockades can be observed in Fig. 4. They will be analyzed in greater detail in the following.

For low bias voltages a Coulomb blockade regime can be observed that extends throughout the whole range of $\varepsilon _d$. Moreover, a strong asymmetry of the Andreev current with respect to the bias reversal is also visible. Such an asymmetry is a direct consequence of the interplay between the magnetic states of the quantum dots and Cooper pairs that are spin singlets. In particular, if two neighboring quantum dots become occupied by electrons of the same spin forming a triplet state, transport to superconductor is blocked as the triplet state does not match the symmetry of the superconductor, while it is not blocked in the opposite direction for tunneling to the normal contact. Of course, in the considered setup the situation is more complex, as we consider the quadruple quantum dot plaquette, however, the mechanism is similar and is associated with occupation of high-spin states that result in asymmetric behavior of the Andreev current.

The asymmetric behavior mentioned above can be clearly observed for both selected values of $\Gamma _{\textrm{CAR}}$. More specifically, one can see that regardless of the parameters, there is a critical value of the positive voltage $V_{\textrm{c}}$, for which a complete blockade of the Andreev current occurs. This blockade is accompanied by a pronounced negative differential conductance, see the bottom row of Fig. 4. By carefully inspecting Fig. 5, which shows the bias dependence of the relevant expectation values, it can be deduced that above the threshold voltage, each quantum dot is occupied by electrons of the same spin, resulting in the $S=2$ state. Because of that, no Cooper pairs can form, and, hence, the Andreev transport is suppressed. This is yet another example of an Andreev current blockade^74–76^, now due to the formation of the spin quintuplet state. This region is marked as Andreev blockade (AB) in Fig. 5.

On the other hand, in the low bias voltage regime, for $\Gamma _{\textrm{CAR}}=t/2$, i.e. below the critical value, there is a pronounced blockade of the Andreev current due to the occupation of three electron spin quadruplet state, which is a signature of Nagaoka ferromagnetism, see Fig. 5c. This region extends over $-1.9 \lesssim \varepsilon _d/t \lesssim -0.9$, and we refer to it as Nagaoka ferromagnetism blockade (NFB). When the bias voltage increases, other states start participating in transport and the blockade due to the Nagaoka magnetism is lifted. For other values of $\varepsilon _d$ shown in the left column of Fig. 4 and out of resonance, in the low bias voltage regime the system’s occupancy is even and characterized by antiferromagnetic order (zero net spin) (cf. Fig. 3c,d). In particular, for $\varepsilon _d$ close to the Fermi level, a single delocalized singlet state is occupied, while for values of $\varepsilon _d$ deep below the Fermi level, four-electron singlet states dominate. Due to well-defined occupancy, the tunneling processes are suppressed.

In the case of $\Gamma _{\textrm{CAR}}$ above the critical value allowing for the Nagaoka ferromagnetism to occur, shown in the right column of Fig. 4, in the low bias voltage regime, the quandruple quantum dot is occupied by spin singlet state. The Andreev current starts flowing once the bias voltage exceeds a threshold voltage. However, similar to the previous case, further increase of positive bias voltage results in the current suppression due to trapping the system in a high-spin state, see the right column of Fig. 5.

It is also important to note that, as can be seen in Fig. 4, the flow of positive current is limited to certain regime of *eV*, which depends on $\varepsilon _d$ and $\Gamma _{\textrm{CAR}}$. In general, increasing $\Gamma _{\textrm{CAR}}$ and moving $\varepsilon _d$ closer to the Fermi level widens the transport window for the positive current. The current in this regime is dominated by high-occupancy states with $S = 1$. Besides that, for the dots’ level energies closer to zero, e.g. $\varepsilon _d/t = -0.5$, there is an additional range of values, for which there is a flow of current, however, with significantly smaller magnitude. Here, the dominating contribution comes from states with $S = 3/2$ and to a lesser extent from $S = 1/2$ states. This results in small current flowing between the metallic tip and the superconductor. In the case of negative current, the situation is more complex. There are multiple excitation lines visible in the differential conductance. Moreover, no strong current suppression is present, as tunneling to the metallic lead is allowed even if quadruple quantum dot is occupied by high-spin states.

## Discussion

In conclusion, we have analyzed the phase diagram and the transport properties of a quadruple quantum dot system subject to superconducting proximity effect. In particular, we have focused on the parameter space when the properties are determined by the competition between the superconducting correlations and Nagaoka ferromagnetism. First of all, we have shown that the phase space of the system consists of spin singlet, doublet and quadruplet phases, depending on the ratio of Coulomb correlations, on-dot pairing amplitude and the hopping between the quantum dots. The quadruplet spin ground state, which is a signature of Nagaoka ferromagnetism, persists even in the presence of superconducting pairing provided the pairing is moderate. When it becomes stronger, the crossed Andreev reflection processes, which favor singlet configuration between neighboring quantum dots, suppress Nagaoka ferromagnetism and eventually lead to the singlet ground state of the system.

To provide more analytical insight into the problem, we then considered the low-energy regime where only CAR processes are relevant, while DAR is negligible due to assumed large Coulomb correlations. In such a case, we examined the phase space as a function quantum dots’ energy level and the strength of CAR processes, revealing parameter space in which the Nagaoka ferromagnetism is present, which is also signaled by ferromagnetic correlations between all quantum dots. The analytical formulas for the critical values of the parameters for the transition between the quadruplet and singlet states have also been provided.

Finally, by employing the real-time diagrammatic technique in the lowest order, we have calculated the Andreev current and the corresponding differential conductance assuming that the system is probed by a lead attached to one of the quantum dots. In such an STM geometry, we found an extended region of current suppression associated with the presence of Nagaoka ferromagnetism. Moreover, we have also uncovered an Andreev current blockade due to the occupation of spin quintuplet states of the system in the nonlinear response regime.

We believe that our results provide further insight into the general properties of hybrid quantum dot systems, shedding light on the interplay between superconductivity and magnetism, in particular in the context of Nagaoka ferromagnetism present in coupled quantum dot structures.

## Methods

The real-time diagrammatic technique is based on the systematic perturbation expansion of the reduced density matrix of the system and the operators in question with respect to the tunneling Hamiltonian^68,70,71^. This implies that the obtained results are relevant in the weak coupling regime $\Gamma \ll k_{\textrm{B}}T$, where $k_{\textrm{B}} T$ stands for the thermal energy of the system. In our considerations, we take into account the lowest-order terms of the expansion, which correspond to sequential tunneling processes, assuming that the contribution of higher-order tunneling processes is much smaller. In other words, we assume that the coupling to normal leads is so weak that any Kondo-like correlations do not arise.

Within the real-time diagrammatic technique, the time evolution of the reduced density matrix on the Keldysh contour can be expressed as a sequence of transitions between the eigenstates of the effective Hamiltonian (7),

21 $$\begin{aligned} H_{\textrm{QDs}}^\textrm{eff} {|{\chi }\rangle } = \varepsilon _\chi {|{\chi }\rangle }, \end{aligned}$$

where ${|{\chi }\rangle }$ denotes the many-body eigenstate of $H_{\textrm{QDs}}^\textrm{eff}$ with eigenenergy $\varepsilon _\chi$. In the stationary state the occupation probabilities of those eigenstates $p_{\chi }$ can be found from the following matrix equation^68,70,71^

22 $$\begin{aligned} {\textbf{W}}{\textbf{P}} = 0, \end{aligned}$$

together with the normalization condition $\sum _{\chi }p_{\chi } = 1$. Here, ${\textbf{P}}$ denotes a vector containing probabilities of the eigenstates ${|{\chi }\rangle }$ and ${\textbf{W}}$ is the corresponding matrix of self-energies, with elements $W_{\chi \chi ^\prime }$ taking into account transitions between the states ${|{\chi }\rangle }$ and ${|{\chi ^\prime }\rangle }$. Having calculated the probabilities of the states ${|{\chi }\rangle }$ it is possible to evaluate the current flowing to the lead $\alpha$ from^68,70,71^

23 $$\begin{aligned} I_{\alpha } = \frac{e}{2\hbar }\textrm{Tr}\left\{ {\textbf{W}}^{I_{\alpha }}{\textbf{P}}\right\} , \end{aligned}$$

where *e* is the elementary charge, $\hbar$ is the reduced Planck’s constant and ${\textbf{W}}^{I_{\alpha }}$ is the modified self-energy matrix accounting for the number of electrons transferred through a given junction.

## Data Availability

The datasets used and/or analysed during the current study available from the corresponding author on reasonable request.
